# Plasmonic propagations distances for interferometric surface plasmon resonance biosensing

**DOI:** 10.1186/1556-276X-6-388

**Published:** 2011-05-17

**Authors:** Dominic Lepage, Dominic Carrier, Alvaro Jiménez, Jacques Beauvais, Jan J Dubowski

**Affiliations:** 1 Department of Electrical and Computer Engineering, Université de Sherbrooke, Sherbrooke, QC J1K 2R1, Canada

## Abstract

A surface plasmon resonance (SPR) scheme is proposed in which the local phase modulations of the coupled plasmons can interfere and yield phase-sensitive intensity modulations in the measured signal. The result is an increased traceability of the SPR shifts for biosensing applications. The main system limitation is the propagation distance of the coupled plasmon modes. This aspect is therefore studied for thin film microstructures operating in the visible and near-infrared spectral regions. The surface roughness of the substrate layer is examined for different dielectrics and deposition methods. The Au layer, on which the plasmonic modes are propagating and the biosensing occurs, is also examined. The surface roughness and dielectric values for various deposition rates of very thin Au films are measured. We also investigate an interferometric SPR setup where, due to the power flux transfer between plasmon modes, the specific choice of grating coupler can either decrease or increase the plasmon propagation length.

## Introduction

Surface plasmon resonance (SPR) is a prominent method widely used for the last two decades [1] in research of label-free characterization and sensing of biological agents, such as viruses and bacteria [2]. To expand the detection capability of SPR, a novel self-referenced interferometric scheme has been proposed to integrate with the SPR architectures. The proposed approach introduces a phase-based signal measurement that complements the classical intensity-based SPR measurement. Multiplexing of those signals leads to an increase precision in the general SPR tracking and thus results in an increased sensitivity of the device.

One of the main limitations of this technique is its reliance on the propagation distance of the coupled SPs (Λ_SP_), as the efficiency of SPR interferometry is directly related to Λ_SP_. For applications in biosensing, this represents an important constraint since SPs are coupled at visible (VIS) or near-infrared (NIR) energies (*E*_SP_) on very thin, typically less than 45 nm, metallic films. Moreover, one side of the metal is necessarily exposed to the probed media, making biosensing SPR interfaces asymmetric. Under those conditions, the long range SPs (LRSPs) cannot be employed. Therefore, we address the fundamental variables influencing SP propagations. The primary aspect is the nanofabrication itself, where the thin films surface roughness is examined for different materials and deposition methods. In addition to the geometry, the dielectric values of the metallic layers are examined as a function of their deposition rates. Finally, a specific configuration of gratings for the SPR interferometry is presented, in which the SPs can couple with an additional SP mode to result in increased propagation distances.

## SPR interferometry

**Figure 1:**
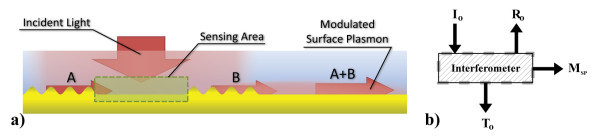
**SPR Interferometer;** (a) Interference of adjacent SP modes; the incident coherent wave couples SP modes on both finite gratings. The probing SP (A) propagates across the cavity and recombines with the reference SP (B), thus forming the combined SP mode (A + B). As the optical path length of the cavity is increased by the presence of biomolecular agents on the surface, the probing SP is phase delayed and the resulting interference pattern will be modified, cycling from constructive to destructive interferences. **(b) **Conceptualization of the system, where an incident light (*I*_o_) hits a grating pair. The light intensity is then distributed between the transmission (*T*_o_), the reflection (*R*_o_), the coupled SPR (*M*_SP_) and some constant absorption. As *M*_SP _is modulated by the phase shift induced by the cavity, monitoring *R*_o _or *T*_o _can reveal information on the interference conditions of A + B.

The basic principle of the SPR interferometry is schematised in Figure 1, where a single coherent beam is used to excite SP modes through spatially localized finite gratings distributed evenly on the metal-dielectric architecture. Those SP modes propagate outwards of the finite grating regions into the cavity regions, where they are phase delayed by an overlying biomolecular environment, before they interfere with the neighbouring SP modes. In a reflection-based SPR experiment, modulations in the reflection (*R*_o_) deliver the information about the SPs interference. In the case of transmission-based SPR, where the illumination source is embedded in the device [3], the transmission (*T*_o_) would be monitored.

**Figure 2:**
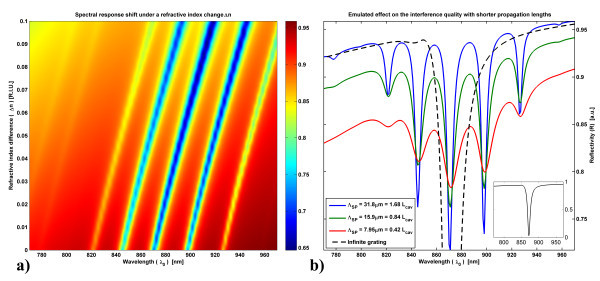
**Far field SPR interferometric signal for an array of grating pairs;(a)** Evolution of the signal under a change of the refractive index. The refractive index of a flat, 250-nm thick, layer overlying the interface is increased by Δ*n *to emulate the increase in the average concentration of a molecular monolayer on top of the gold surface. Thicker layers would induce a steeper shift in λ_o_. For the presented case, Λ_SP _= 31.8 μm. **(b) **Effect of the propagation lengths on the interference fringes' signal quality. The shift of the curves baseline is due to the simulated increase in the metal absorption. The dotted black curve (fully shown in inset) presents the infinite grating scenario: more power is coupled to the surface and no interference is visible, as there are no cavities.

To demonstrate the ability of the SPR interferometric architecture to produce a multiplexed signal, finite element method (FEM) simulations were carried out using COMSOL Multiphysics™ v3.5a software in conjunction with Matlab^®^, expanding from the results reported in [4] by increasing the number of finite gratings. The resulting multiple interference increases the measured signal's quality factor. The results presented in Figure 2 were simulated for a semi-infinite flat interface of Au and air, with a regular array of finite gratings, evenly separated and illuminated as in Figure 1. The 20-nm high sinusoidal gratings have a periodicity of *P*_G _= 805 nm, are 8.57 μm in length (10⋅λ_SP _at 1.4251 eV) and are spaced by 18.85 μm (22⋅λ_SP _at 1.4251 eV), where λ_SP _denotes the wavelength of the SPs. The incident light ranges from 1.28 to 1.61 eV (λ_o _= 770 to 970 nm) and is normally incident to the surface. Figure 2a illustrates the dependence of the reflected (*R*_o_) SPR interferometric signal on the changes in the refractive index of a 250-nm thick layer deposited atop of the investigated microstructure. In this figure, the number of traceable SPR intensity minima is multiplied by the interferences fringes. By the central limit theorem in statistical theory [5], the precision of the absolute SPR shift is increased by *N*^1/2^, where *N *denotes the number of interference fringes. The number of interference fringes is directly proportional to the cavity's optical length bounded by Λ_SP_. The fringes measurability (intensity vs. background) is also a function of Λ_SP_. This is shown in Figure 2b, where the impact of Λ_SP _on the interference signal quality is depicted. As presented, the propagation length has a severe impact on signal quality for a specific architecture, as a shorter propagation length leads to a larger difference in amplitude between two SP modes interfering. This difference reduces the interferometric signal's amplitude in relation to the background reference, resulting in a reduced S/N ratio. To make use of the SPR interferometry for biosensing, the SPs propagation distance should be as long as possible.

LRSPs have already been studied extensively [6]. Though they present advantageous properties for integrated plasmonics, self-coherent LRSPs are by nature incompatible with biosensing applications: they are either entrapped in dielectrics layers, supported by thick bulk substrates, or have low energies in the IR. Given the decreasing slope in *ε *of biochemical materials versus energy [7], the sensitivity of SPR is strongly diminished for IR. Therefore, more traditional means have to be considered when trying to increase the propagation lengths of SPs while taking into account practical issues for biosensing, such as an open metallic surface, thin-films and SPR at VIS-NIR energies (higher energies damaging the samples while lower energies present poor sensitivities). The first and most practical aspect to consider is the nanofabrication itself.

## Nanofabrication and roughness

The SPs propagation distances are limited by thermal losses in the metal at a given energy *E*_SP_(*ω*). Additional losses will occur through radiations in thin films, illustrated by a larger SPs complex wave vectors due to coupling to the other interface (known as Fano modes) [8]. The surface roughness is also known to play a very important role in the limitation of the SPs propagation distances, as the corrugation will diffract a fraction of the SPs light flux. Indeed, the mean free path of the SPs wave has been found to be inversely proportional to the square of the surface roughness height, for a given SP energy and fixed metal dielectric (the complete formulation is available in [9]). The fabrication of SPR devices to be employed in the VIS-NIR range of energies has become possible in the last decades due to the improved fabrication methods at the nanoscale. Nonetheless, the surface roughness of the films and nanostructures now has a larger impact on SPs modulated signals, as the geometrical structures have sizes comparable to the inherent roughness of the employed fabrication methods. For example, in Figure 1 the grating has a line height of 20 nm, but the grain size of e-beam evaporated Au is around 6 nm. The most straightforward way of increasing the SPs propagation length for SPR interferometry is to reduce the surface roughness to a minimum during the fabrication process of the architectures. In many SPR experiments, a dielectric layer has to be fabricated on the top of a functional substrate such as a semiconductor. This is the case for transmission-based experiments [3] or for reflection-based experiments in which active components are involved and where one side of the metal film is bounded by a deposited dielectric [10-12].

**Figure 3:**
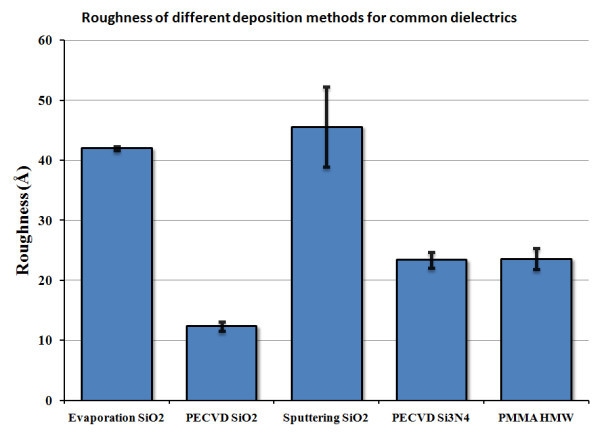
**Surface roughness for various dielectric materials and fabrication methods, as measured by ellipsometry.** Uncertainties represent the standard variation between three independent material depositions [13].

To reduce the roughness, we analysed different materials and deposition techniques. The substrate layer on which the metallic layer is going to be deposited is the first concern, as its roughness will directly impact the quality of the subsequent thin films. All the films studied were deposited on Si substrate, whose surface roughness is below 8 Å under AFM. Figure 3 presents the surface roughness, measured by ellipsometry, for three dielectrics commonly employed in nanofabrication [13]. SiO_2 _is initially studied, for which three different fabrication methods were explored: e-beam evaporation, plasma sputtering and plasma-enhanced chemical vapour deposition (PECVD). Si_3_N_4 _is a good candidate, due to its relatively large dielectric constant, and was deposited through PECVD. The spin coating of a common electro resist, polymethyl methacrylate (PMMA), is also presented. On average, 300 nm of SiO_2 _or Si_3_N_4 _and 150 nm of PMMA were deposited atop Si substrates. Figure 3 shows that SiO_2 _deposited through PECVD is the best candidate for thin films in the present case, with a consistent surface roughness of 12.3 ± 0.8 Å. The energy-dependent dielectric values for the resulting layers have been measured by ellipsometry and are presented elsewhere [13]. SEM and AFM measurements were also carried, concurred with the presented results, but are not exposed here for clarity.

**Figure 4:**
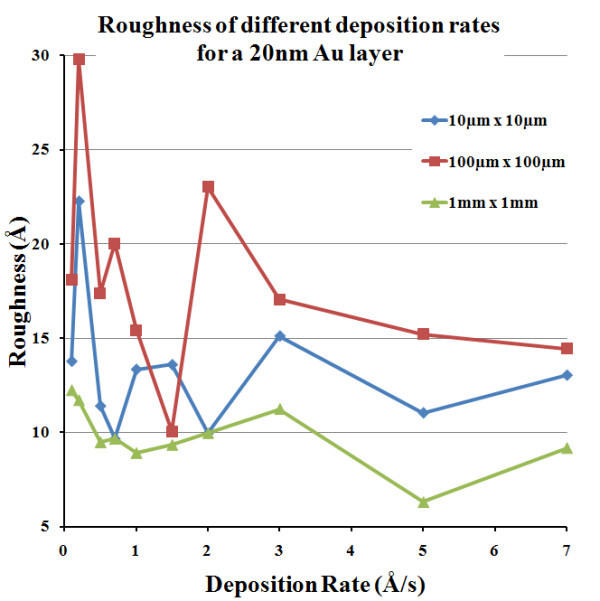
**Surface roughness for various e-beam deposition rates of Au**. Various sampling areas were examined: the 10 and 100 μm^2 ^regions are measured by AFM while the 1 mm^2 ^region is the roughness yielded by ellipsometry [13].

**Figure 5:**
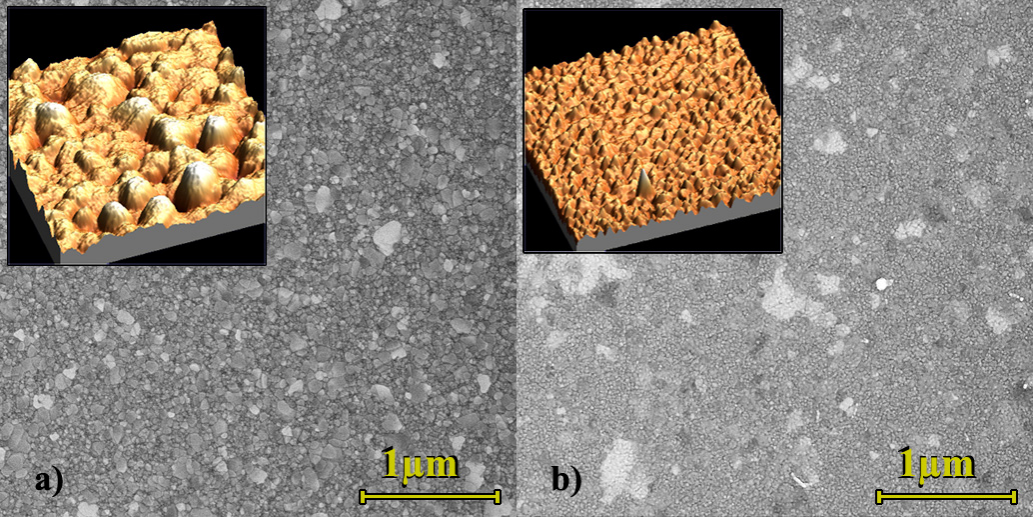
**SEM images of surface roughness for of Au surfaces;** as presented in [13]for **(a)** 0.2 Å/s deposition rate. The roughness is high and but relatively homogeneous over the surface. Au grains are clustering over the surface and present a lower density. The inset is a 10 μm^2 ^AFM profile. **(b) **At 0.7 Å/s deposition rate, the localized surface roughness is smaller, more compact and a lower clusterization with the typical grain size of Au at 6 nm.

The successive layers for Figure 1 structure consist in a continuous thin film of Au atop of which a grating region is constructed for the SPs coupling. Again, the surface roughness of Au is studied, this time only for the e-beam evaporation technique (using a BOC Edwards evaporator model Auto 306) for various deposition rates. The target thickness for the Au layers is 20 nm. Figure 4 presents the surface roughness for the various deposition rates [13]. In depth SEM analyses have shown that for small deposition rates (≤1 Å/s), Au nanodroplets tend to cool down and form 100-200 nm wide clusters, thus yielding a relatively high surface roughness. On the other hand, for large deposition rates (>3 Å/s), the Au grains remain small (approximately 6 nm) and are very compact on the surface. However, very large Au pieces, up to about 1 μm^2^, are found in this case on the surface. Examples of these two behaviours are presented in Figure 5. As shown in Figure 4, a middle value for the deposition rate, at around 1 Å/s, presents tradeoffs of the two regimes and seems to be the ideal case for deposition of low-roughness Au films. Au-plated quartz substrates commercially available have been measured to have a roughness around 40 Å, making them less suited for long range SPs experiments or to achieve narrow SPR peaks. To conclude on surface roughness, we can estimate that our worst case would consists of sputtered SiO_2 _with a 0.2 Å/s deposition rate, yielding a 55 Å surface roughness while the best case scenario, made of a PECVD SiO_2 _layer with a Au layer evaporated at 1.5 Å/s, would yield a surface roughness of 15 Å. From these numbers, we can estimate that at a given energy, the contribution of surface roughness to SPs loss in scattering is reduced by a factor of 13 × [9]. Well-known smoothing methods, such as thermal annealing, are generally incompatible with thin film technology. Indeed, heating thin Au films (<50 nm) increases the formation of larger clusters, grains or flakes [14-16], which can be useful for some applications [16], but not for planar SPR where propagating SPs would scatter. Therefore, ab initio precautions have to be taken to generate very thin and flat metallic layers.

**Figure 6:**
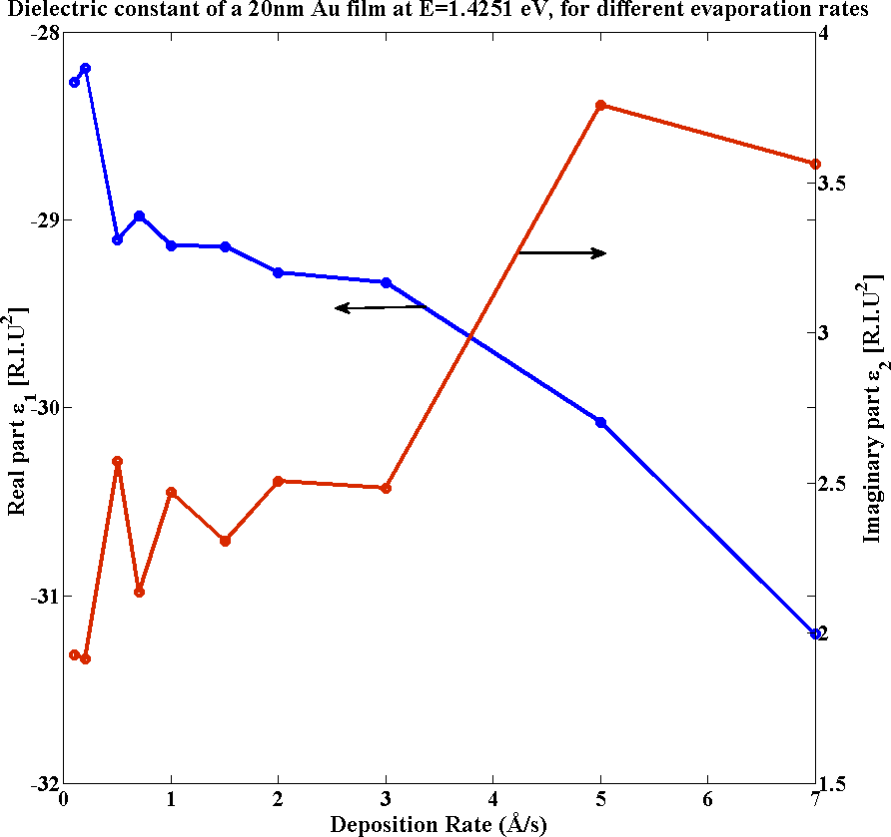
**Real and imaginary part of the 20-nm Au film at *E *= 1.4251 eV for various deposition rates**. As the film compactness increases, the values tend towards bulk constants.

Another fabrication aspect to take into account is the value of the dielectric constants of the films, especially those of the metal layer. These values were measured for various energies by ellipsometry for the thin Au films deposited at various rates. The results for *E *= 1.4271 eV are presented in Figure 6. As one can observe, both real and imaginary parts of the dielectric constant, *ε*_Au_, are increasing with the deposition rate. This can be understood by analysing the AFM and SEM results showing that the film density increases with the deposition rate: thus, a higher value of the effective dielectric constant approaching that of the bulk material.

To estimate the propagation lengths of the surface plasmon modes, we have factored in our simulations the measured experimental dielectric properties of the metallic substrate as well as the underlying structure. A finite incident beam is employed to excite the SP mode within a specific region; the propagating mode's EM field intensity decay is observed outside of that region and fitted with a decay model using non-linear regression, to extract the mean free path Λ_SP_. To isolate the effect of the dielectrics values, the thin films are considered to have perfectly flat surfaces on both sides. The SPs propagations for these simulations are therefore limited by losses to radiations coupling (to Fano modes) and by electron dampening (thermal loss), but there is no scattering into free space. The increase in experimentally measured dielectric values of the thin films, real and imaginary, induce an overall increase of the SP propagation lengths. The Λ_SP _on the flat 20 nm layer can increase from 4.69 ± 0.02 μm for the 0.5 Å/s layer (with *ε*_Au _= -29.1032 + 2.5736i) to 5.22 ± 0.02 μm for the 7 Å/s (with *ε*_Au _= -31.2071 + 3.5632i), a 10% increase. The film with a larger dielectric constant, despite having greater thermal losses to electron damping, results in a better SP mode confinement. This effect would be comparable to increasing the film thickness, reducing the radiation leaks through coupling to the other interface, lowering the SP wave vector and increasing the propagation lengths.

## Surface plasmon mode coupling

**Figure 7:**
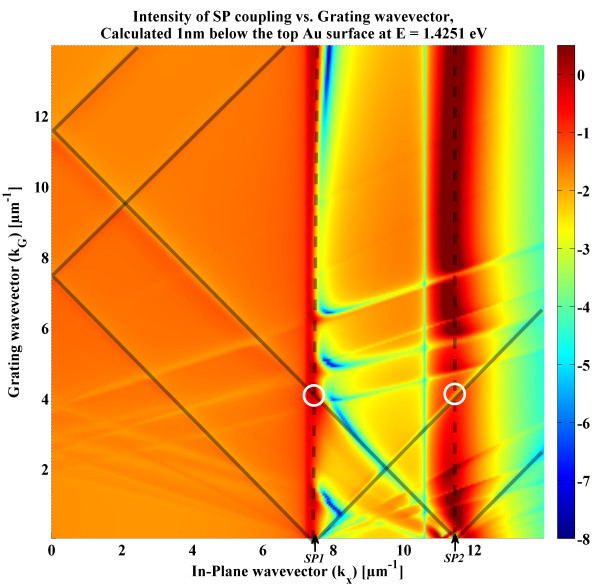
**Log of the intensity of SPs coupling versus the grating wave vector *k*_G _for the architecture of Figure 1; at the 0th diffraction order**. SP1 is coupled at *k*_*x *_= 7.42 μm^-1^. SP2 is coupled at *k*_*x *_= 11.48 μm^-1 ^and weakly perturbed by the surface changes. Lines of SPs coupling though the grating are visible. Coupling through the ±1st diffraction order is highlighted by the semi-transparent lines. Higher orders of diffraction and coupling (parallel and anti-parallel) are visible in the graph (±2nd, ±3rd, etc), but are not underlined for clarity purposes. A practical application for Λ_SP _is found at *k*_G _= Δ_SP_, shown by the white circles. A cross section of Λ_SP _at *k*_*x *_= *k*_SP1 _is shown in Figure 8.

**Figure 8:**
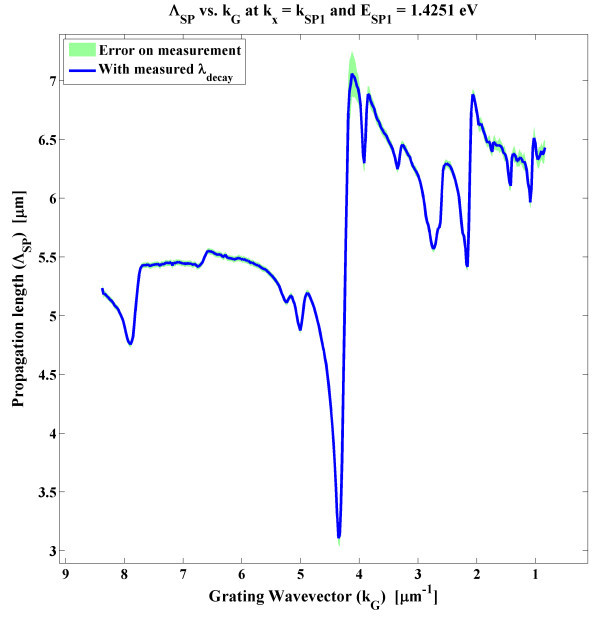
**Λ_SP _at *k*_SP1 _for various *k*_G_**. The various peaks and dips are attributed to the different SPs mode coupling shown in Figure 7. A maximum in Λ_SP _is found when *k*_G _= Δ_SP_.

In addition to the fabrication methods, specific designs of the interferometric architecture can help to increase propagation lengths. As detailed widely in literature, SP modes can be coupled on both interfaces of thin film architectures, i.e. on the surface and below the metal [8,17]. Simultaneously, coupling both SP modes, SP1 atop the thin film and SP2 under, opens a plethora of luminous flux exchange phenomena [8]. When coupling SPR through a grating, as in Figure 1, several coupling events can occur between SP1 and SP2, as a function of the chosen grating periodicity, *P*_G_. Figure 7 presents the EM-field intensity distribution, calculated 1 nm below the metal layer, as a function of the in-plane wavevector *k*_*x *_and the grating wavevector *k*_G _= 2π/*P*_G_. The intensity shown is only for the 0th diffraction order of the grating, i.e. simple transmission, for clarity. The lines illustrate the effects of the grating's diffraction on the 0th order intensity distribution. At the SP wavevectors *k*_SP1 _and *k*_SP2_, the peaks and drops in intensity correspond to various SPs flux exchange. Anti-parallel coupling phenomena arise when forward (+) and backward (-) propagating SPs are coupling. Thus, SP1^+ ^can couple with SP1^- ^at *k*_G _= 2|*k*_SP1_|/*n*, SP2^+ ^with SP2^- ^at *k*_G _= 2|*k*_SP2_|/*n *and SP1^+/- ^with SP2^-/+ ^at *k*_G _= (|*k*_SP1_| + |*k*_SP2_|)/*n*, where *n *is the diffraction order. More interesting are the parallel coupling between SP1 and SP2 travelling in the same directions, which occurs when *k*_G _= Δ_SP_/*n*, with Δ_SP _= |*k*_SP1_| - |*k*_SP2_|. The parallel coupling between SP1 and SP2, through the first diffraction order *n *= 1, is of specific interest as it increases the propagation distance of the SPs on the surface. The propagation distance of SP1 for various *k*_G _is presented in Figure 8, where an increase by a factor of 1.5 can be achieved when *k*_G _= Δ_SP_. The SP1 and SP2 modes can optically pump each other and thus combine in a hybrid guided mode, which have been studied in the literature [8]. The sensing response still comes from the reflected (or transmitted) incident light, which is modulated by the phase shift induced by the cavity. Therefore in Figure 1, the incoming light ray can directly inject SPs at *k*_SP1_, which in turn can couple through the grating with SP2 by *k*_SP2 _= *k*_SP1 _+ Δ_SP_. The resulting guided SP mode is propagating on both interfaces and does so much further. This specific selection of grating can then be employed for SPR interferometry and increase its sensitivity.

## Conclusion

The presented SPR interferometry method is a relatively straightforward way of enhancing the sensitivity of classical intensity-based SPR biochemical sensing, by introducing SPs phase modulations in the measurements. The number of traceable SPR peaks is multiplied by the SPs interference and tracking those multiplied SPR peaks enable a better resolution on the absolute value of surficial SPR shift. The main limitation of the method is its dependence on the SPs propagation distance Λ_SP_.

We have therefore examined the principal factors influencing Λ_SP _in experimental setup for biosensing, which simply consists of a thin film Au layer atop a dielectric, measured in the VIS or NIR regions. The results can apply to various architectures, including Kretschmann-Raether setups.

The initial focus was on surface roughness, playing an important role in thin film SP propagation. We found that a careful optimization of the fabrication process can reduce the SP loss due to quasi-random diffractions by a factor of 13 ×. The resulting films have dielectric values dependent on their deposition rates, which obviously plays a role in the SP wave confinement, and thus its Λ_SP_. Finally, it was shown that the periodicity of the selected grating can have important impacts, negative and positive, on Λ_SP_. Various SP modes (or more precisely Fano modes) can be coupled in parallel and anti-parallel behaviours. The specific parallel coupling between SP1 and SP2 through the first diffraction order of the grating has been found to increase the propagation lengths by a factor of 1.5 in the SPR interferometer, enhancing the sensitivity of the method even further.

By carefully addressing the presented aspects, we conclude that SPR interferometry is experimentally feasible and has the potential to increase SPR sensitivity by a factor proportional to the SPs propagation distances, Λ_SP_.

## Abbreviations

AFM: atomic force microscopy; FEM: finite element method; LRSPs: long range SPs; NIR: near-infrared; PECVD: plasma-enhanced chemical vapour deposition; PMMA: polymethyl methacrylate; SPR: surface plasmon resonance; VIS: visible.

## Competing interests

The authors declare that they have no competing interests.

## Authors' contributions

DL carried out the main conception and design of the SPR architectures, participated in the analysis and interpretation of data, did the calculations for plasmon mode coupling and drafted the manuscript. DC carried the COMSOL simulations and participated in the analysis and interpretation of data. AJ designed the experiments and carried the nanofabrication of the samples. JB and JJD have given final approval of the version to be published. All authors read and approved the final manuscript
